# Effective suppression of Dengue fever virus in mosquito cell cultures using retroviral transduction of hammerhead ribozymes targeting the viral genome

**DOI:** 10.1186/1743-422X-6-73

**Published:** 2009-06-04

**Authors:** Pruksa Nawtaisong, James Keith, Tresa Fraser, Velmurugan Balaraman, Andrey Kolokoltsov, Robert A Davey, Stephen Higgs, Ahmed Mohammed, Yupha Rongsriyam, Narumon Komalamisra, Malcolm J Fraser

**Affiliations:** 1 Department of Biological Sciences, Eck Institute of Global Health, University of Notre Dame, Notre Dame, Indiana 46556, USA; 2 Department of Medical Entomology, Faculty of Tropical Medicine, Mahidol University, Bangkok, Thailand; 3 Department of Microbiology and Immunology, University of Texas Medical Branch, Galveston, Texas, 77555, USA; 4 Department of Pathology, Center for Biodefense and Emerging Infectious Diseases, University of Texas Medical Branch, Galveston, Texas, 77555, USA

## Abstract

Outbreaks of Dengue impose a heavy economic burden on developing countries in terms of vector control and human morbidity. Effective vaccines against all four serotypes of Dengue are in development, but population replacement with transgenic vectors unable to transmit the virus might ultimately prove to be an effective approach to disease suppression, or even eradication. A key element of the refractory transgenic vector approach is the development of transgenes that effectively prohibit viral transmission. In this report we test the effectiveness of several hammerhead ribozymes for suppressing DENV in lentivirus-transduced mosquito cells in an attempt to mimic the transgenic use of these effector molecules in mosquitoes. A lentivirus vector that expresses these ribozymes as a fusion RNA molecule using an *Ae. aegypti *tRNA^val ^promoter and terminating with a 60A tail insures optimal expression, localization, and activity of the hammerhead ribozyme against the DENV genome. Among the 14 hammerhead ribozymes we designed to attack the DENV-2 NGC genome, several appear to be relatively effective in reducing virus production from transduced cells by as much as 2 logs. Among the sequences targeted are 10 that are conserved among all DENV serotype 2 strains. Our results confirm that hammerhead ribozymes can be effective in suppressing DENV in a transgenic approach, and provide an alternative or supplementary approach to proposed siRNA strategies for DENV suppression in transgenic mosquitoes.

## Background

Dengue viruses (DENV); (*Flaviviridae*), etiologic agents of dengue fever (DF) and dengue hemorrhagic fever/dengue shock syndrome (DHF/DSS), are transmitted to human populations by the mosquitoes *Aedes aegypti *and *Ae. albopictus*. An estimated 50–100 million cases of DF are reported each year, with 500,000 cases of DHF/DSS and more than 20,000 deaths [1]. Several factors that contribute to the emergence of this disease complex include the collapse of mosquito vector control, the demise of public health programs, mosquito drug resistance, climatic changes, expanding urbanization and increased global travel and commerce [2,3]. While promising vaccine candidates are undergoing clinical trials [4], these vaccines will not be available for general use for quite some time.

Alternative strategies targeting DENV in mosquito cells and tissues have demonstrated some promise for suppression of the virus in mosquito vector populations. Modified antisense oligonucleotides [5], induction of RNA interference (RNAi) using both preM-derived sense and antisense encoding sequences expressed from dsSIN virus vectors [6,7] and hairpin dsRNA to mediate RNAi in both mosquito cells [8,9] and transgenic mosquitoes [10,11] have each provided significant levels of DENV suppression.

While RNAi may be an effective mechanism to interrupt viral infection, it also has several potential limitations. Targeted sequences must be at least 21 nt in length limiting the number of target sequences that are conserved among all DENV strains, and escape mutants can result from a single point mutation among the 21 nt of target sequence [12]. RNAi requires a relatively large amount of dsRNA to be effective against viral replication [13], and some viruses may replicate faster than the ability of the RNAi response to suppress the virus [3]. A number of plant and animal RNA viruses effectively escape the RNAi response by encoding proteins that suppresses RNA silencing [10,14,15]. Flaviviruses, in particular, seem to evade the RNAi response by sequestering their replication complex inside a double-layered membrane complex [11].

In an attempt to overcome some of these limitations of RNA-based effector strategies, our lab has focused efforts on RNA-enzyme (ribozyme) mediated viral suppression. In this report we explore the utility of a genetic approach utilizing hammerhead ribozymes (hRz) for suppression of DENV in mosquito cells. hRz can inhibit the replication of a number of RNA viruses including human immunodeficiency virus (HIV; [16,17], hepatitis B virus (HBV; [18,19] and hepatitis C virus (HCV; [20]. These molecules are capable of identifying targets as small as 15 nt in length, potentially allowing highly conserved sequences to be the focus of attack.

In this report we transduced *Ae. albopictus *(C6/36) cells with pantropic retroviral vectors, each expressing one of 14 anti-DENV hRz driven from the *Ae. aegypti *tRNA^val ^promoter. These ribozyme-transduced cells were challenged with virus and assayed for productivity. Northern analyses, immunofluorescence assays, and quantitative real-time PCR demonstrate that C6/36 cells expressing several hRzs were able to suppress DENV replication by at least 75%, with four of these hRzs providing 90 to 99% suppression. Several of these targeted sequences are highly conserved among DENV serotypes, and may facilitate the application of this approach to transgenic mosquitoes.

## Results

### Construction of retroviral transducing vectors expressing anti-DENV hRzs and establishment of transduced C6/36 cells

**Figure 1:**
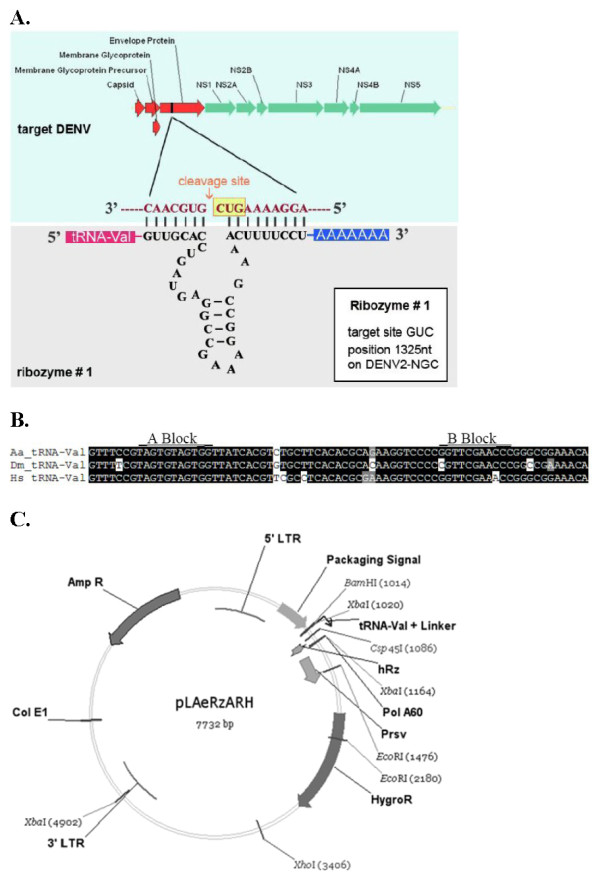
**A: Representative hRz structure and its DENV target sequence**. hRz # 1 nucleotide sequence and structure is depicted. Nucleotides flanking the cleavage site (yellow box) in the envelope protein region of the DENV-2 target RNA are enlarged. The ribozyme cleaves the target RNA at the GUC triplet site following antisense recognition and base pairing of the two ribozyme arms. B: Nucleotide alignments of the Human (Hs), *D. melanogaster *(Dm), and *Ae. aegypti *(Aa) tRNA^val^. The position of the concensus internal A and B blocks of the RNA pol III promoter are indicated. C: Plasmid pLAeRzARH was derived from pLXRN as described in Materials and Methods. The RSV promoter was added to drive independent expression of the hygromycin resistance gene. Expression of each hRz is driven by the tRNA^val ^internal RNA pol III promoter to optimize expression and translocation of the hRzs to the cytoplasm, and a stretch of 60As is attached to the 3' end of the hRz sequence for recruitment of RNA helicase.

hRz are small ribonucleic-based enzymes that are capable of catalyzing target RNA cleavage in a sequence-specific manner. Their mechanism of action involves the pairing of the 5' helix I and 3' helix III arms of the hRz to complementary 3' and 5' base pairs, respectively, on the target RNA (Fig. 1A). The catalytic core of the hRz, or helix II, is responsible for cleavage at a 5'-NUH-3' triplet site on the target RNA, where N can be any of the four nucleotides and H can be A, C or U [21]. Factors that contribute to the success of hRzs as effector genes include (i) high concentration and stability of the hRz within the cellular environment, (ii) colocalization of the hRzs to the target RNA [22,23] and (iii) accessibility of the hRzs to the cleavage site within the context of RNA secondary structure [24]. The addition of a tRNA^val ^pol III promoter upstream of the hRz-coding sequence ensures high production of hRz transcripts and facilitates their transport to the cell cytoplasm [25], overcoming the rate-limiting step of the hRz cleavage mechanism.

We identified the *Ae. aegypti *tRNA^val ^sequence from the GenBank database based upon homology to the *Drosophila melanogaster *tRNA^val ^sequence. The presumptive *Ae. aegypti *tRNA^val ^(GenBank accession: CC142986), shared a 95% similarity (e = 5 × 10^-27^) to the *D. melanogaster *tRNA^val^, including both internal promoter sites (Fig. 1B). This sequence was PCR amplified from *Ae. aegypti *genomic DNA and placed into a pLXRN vector upstream of inserted hRz sequences as detailed in Materials and Methods. A stretch of 60 adenylic acids (A) was linked downstream from the hRz sequences to enhance their catalytic activity by interacting with intracellular RNA helicases [26,27] and improving access to target sites on the viral genome (Fig. 1C).

**Figure 2:**
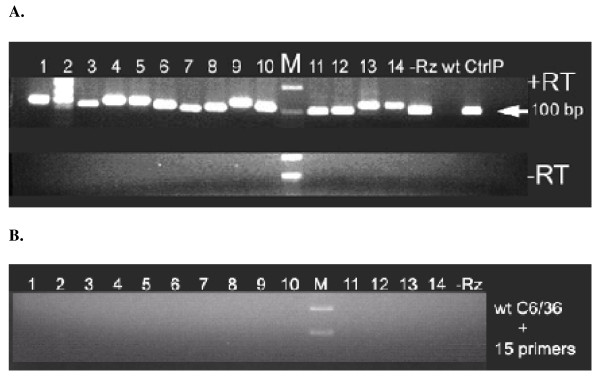
**RT-PCR of total RNA extracted from hRz-C6/36 cells**. (A) hRz expression in the cells was detected by the presence of an hRz-specific band at about 100 bp. Primers for each hRz-C6/36 cells were specific to the hRz insert except for the control lacking a hRz sequence (-Rz) for which the primers were specific to tRNA^val ^and poly(A) tail. (B) Wild-type C6/36 cells failed to give a PCR product when tested with 15 sets of primers (each primer was specific to each hRz). *1–14*: 14 different hRz-C6/36 cells; *-Rz*: C6/36 cells without the hRz insert; *wt*: wild-type C6/36 cells; *CtrlP*: plasmid DNA control for PCR amplification; *M*: 1 Kb Plus DNA ladder; *+RT*: reactions with reverse transcriptase; *-RT*: reactions without reverse transcriptase.

Fourteen ribozyme-encoding retroviruses and one control lacking a ribozyme sequence were used to transduce wild-type C6/36 cells by infecting at an MOI of 30 as described in Materials and Methods. Retrovirus-infected C6/36 cells were placed under hygromycin selection for 4–8 weeks and then analyzed for hRz expression by RT-PCR of total cellular RNA (Fig. 2). Only cells that are transduced will have integrated provirus cDNA transcribing the hRz RNA. Therefore, we can be certain that the detected RNA is not residual lentivirus genomic RNA.

### CPE of DENV infection in the hRz-transduced C6/36 cells

**Figure 3:**
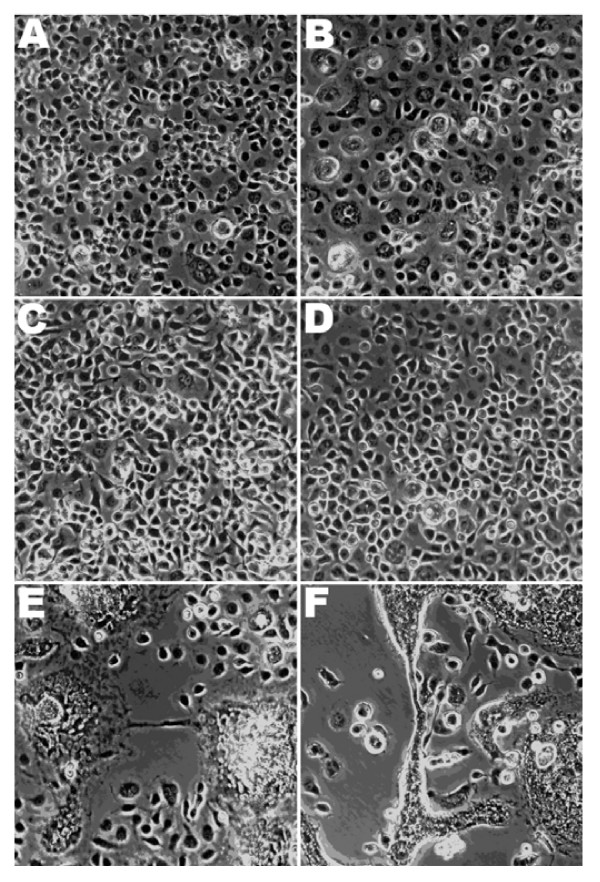
**CPE due to DENV infection of C6/36 cells at 5 dpi**. Images were taken at the 40× magnification. Cells were those transduced with hRz-encoding retroviruses and selected in hygromycin for stable integration of the transgene. Representative infected cell cultures are shown. These are cells transduced with (A) *hRz # 2*, (B) *hRz # 5*, (C) *hRz # 7*, (D) *hRz # 11*, (E) *No Rz *(transduced with lentivirus vector lacking a hRz) or (F) non-transduced *C6/36 *cells.

The CPE of DENV-2 NGC infection in C6/36 cells, characterized by syncytium formation and decreased cell proliferation, was clearly visible 5 days post infection (dpi). Those cells expressing certain hRz exhibited a clear reduction in CPE at 5 dpi, allowing them to grow to confluency, while cells that lack hRz, (i.e. No-hRz and wild-type), exhibited the expected CPE (Fig. 3). The most effective hRz constructs were those that appeared to completely suppress CPE. These were hRz-C6/36 cell lines # 2, 5, 7 and 11.

### Northern analyses for DENV genome

**Figure 4:**
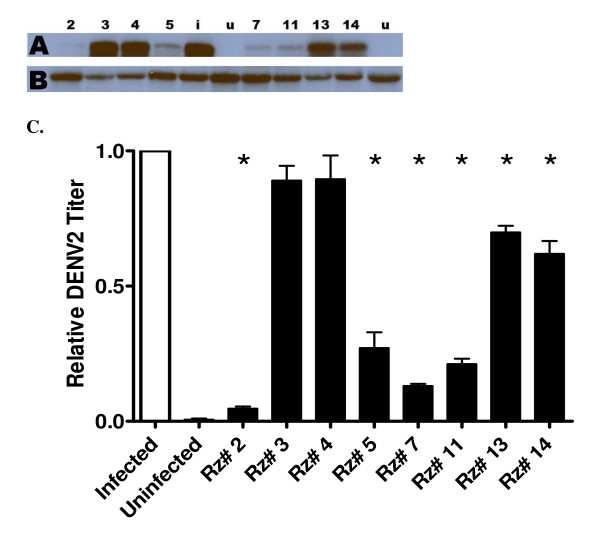
**Northern hybridization analysis of DENV-2 replication in cells expressing hRz constructs**. (A) Total RNA samples hybridized with DENV-specific probes. (B) Actin RNA from the same samples hybridized with a β-actin-specific probe. Each construct is indicated by numbers; *i*: wild-type C6/36 infected with DENV; *u*: uninfected wild-type C6/36. The autoradiograph was exposed for 6 hr prior to developing. (C) Quantification of relative DENV-2 RNA levels from the Northern blot analysis. The scanned autoradiograph was processed in ImageJ software and the relative amount of DENV-2-specific RNA in each sample was compared against that of infected wild-type cells using an ANOVA test (GraphPad Prism 3.0). Statistically significant differences relative to the Infection control (Dunnett's post-test, p < 0.01) are indicated with asterisks. *Infected*: infected, non-transduced C6/36 cells; *Uninfected*: uninfected, non-transduced C6/36 cells; *Rz #*: Different infected hRz cells.

Those transduced cultures that gave at least moderate CPE suppression were analyzed by Northern blot with DENV-specific probes to determine the impact of hRz expression on DENV RNA replication. Infected and uninfected wild-type C6/36 cells were included as positive and negative controls, respectively, with *β*-actin serving as an internal hybridization and loading control. Autoradiographs (Fig. 4A and 4B) were scanned and analyzed by densitometry to estimate the relative amounts of DENV RNA in each sample.

The rapid degradation of ribozyme cleavage products coupled with the very effective suppression of DENV replication in the transduced cells, made detection of hRz cleavage product RNAs difficult by Northern blots. The efficacy of the hRzs was estimated by comparing the relative amount of the target DENV RNA to the infected and uninfected C6/36 control cultures (Fig. 4C). These analyses confirm that hRz-C6/36 cell lines # 2, 5, 7 and 11 suppressed the replication of DENV by at least 25% relative to the infected wild-type cells.

### *In vitro *analyses of hRz cleavage activity

**Figure 5:**
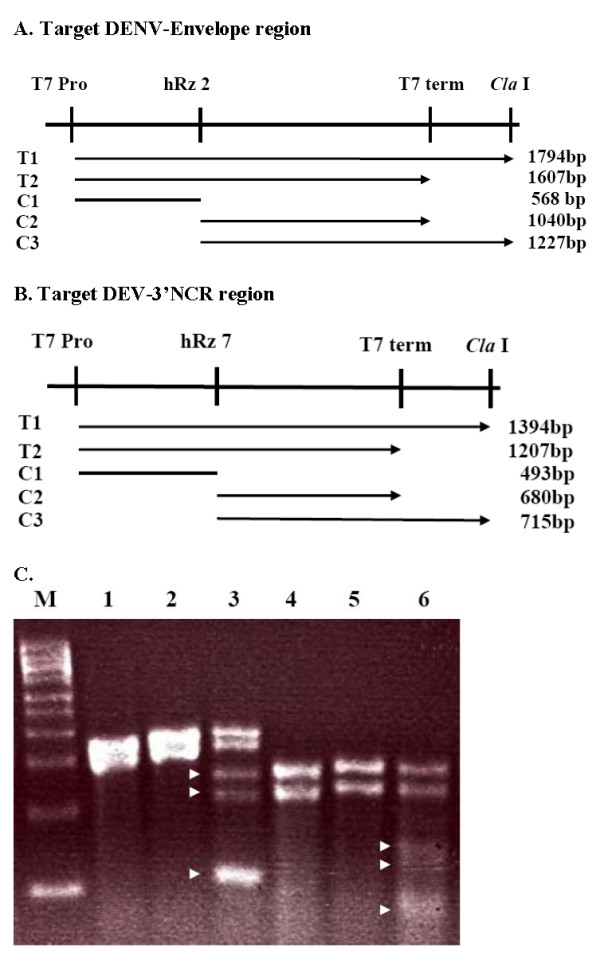
**Confirmation of cleavage activities for representative hRzs**. (A and B) Maps showing the *in vitro *transcripts generated from the linearized pET11a vectors for the hRz # 2 (A) and hRz # 7 (B) target substrates. Positions of the T7 promoter (T7-Pro), hRz-T7 cleavage site (hRz7), T7 terminator (T7 Term), and *Cla *I site used for linearization (*Cla *I) are indicated. T1 and T2 show the extent of two transcripts that are generated in the *in vitro *transcription reaction for each substrate. C1 shows the extent of the single 5' cleavage product from both transcripts from each substrate, while C2 and C3 show the extent of the two 3' cleavage products generated from the two different transcripts produced from each substrate. (C) Agarose gel of *in vitro *cleavage reaction products. *In vitro *transcribed targets and their respective hRz # 2 and hRz # 7 were incubated for 30 min at 37°C. Cleavage products were separated in 2% agarose gels stained with ethidium bromide. Lane M: Millenium™ RNA Marker. Lanes 1–3: *In vitro *transcribed DENV-ENV region target without MgCl_2 _(lane 1), hRz # 2 and without MgCl_2 _(lane 2), and hRz # 2 with MgCl_2 _(lane 3). Lanes 4–6: *In vitro *transcribed 3'NCR region target without MgCl_2 _(lane 4), hRz # 7 without MgCl_2 _(lane 5), and hRz # 7 with MgCl_2 _(lane 6). Arrows in lanes 3 and 6 show the expected hRz # 2 and hRz#7 cleavage products, respectively.

Because the Northern analyses did not allow detection of ribozyme cleavage products, we tested the four most effective ribozymes for their cleavage activity *in vitro*. DNA molecules encoding each hRz construct, including the tRNA^val ^and polyA tail, were synthesized downstream of a T7 promoter sequence, cloned, and expressed *in vitro *as described in Materials and Methods. These *in vitro *transcribed ribozymes were combined with *in vitro *transcribed target RNA molecules containing extensive regions of the DENV-2 NGC genome that encompass hRz # 2 and 5, or hRz # 7 and 11 cleavage sites. The results for two of these ribozymes, hRz # 2 and # 7, are presented in Fig. 5. The cleavage products and hRzs are apparent as distinct bands in the lanes corresponding to each reaction. A third band of unknown identity was detected in each experimental lane as well. We believe this extra fragment is the result of alternative cleavage of the target RNA since the size of the hRz transcripts (50 nt) are too small to appear on these gels, and because these fragment do not appear in the control lanes lacking hRz.

### TCID_50 _immunofluorescence assays

**Figure 6:**
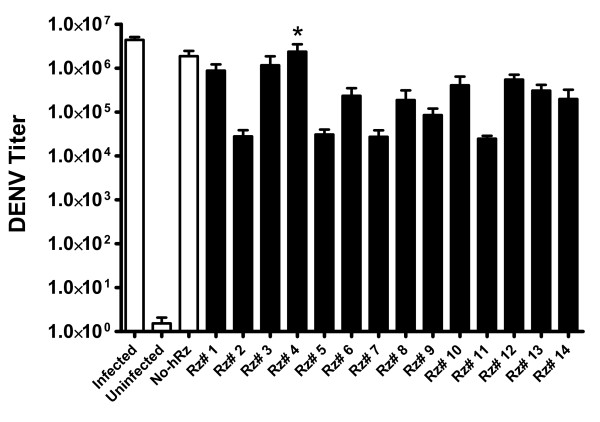
**TCID_50 _immunofluorescence assay**. At 4 day dpi, cells were fixed and stained. Primary antibody against ENV protein and biotinylated-secondary antibody and streptavidin were employed as a fluorescence detection system. A one-way ANOVA test was performed using GraphPad Prism 3.0 software. Asterisk indicate no significant differences relative to the Infected control (Dunnett's, p < 0.01). *Rz # 1–14*: 14 different infected hRz cells; *No Rz*: infected C6/36 cells transduced with the lentivirus vector lacking a hRz insert.

We determined the effectiveness of each ribozyme in suppressing overall infectious virus production using an immunoflourescence-based TCID_50_assay. Cell culture medium collected at 4 dpi from infected cells was assayed as described in Materials and Methods using a DENV envelope protein-specific monoclonal antibody. While all ribozyme transformed, and even the No-hRz transformed control cells, exhibited statistically significant reductions in overall infectious virus production, hRz-C6/36 cell lines # 2, 5, 7 and 11 had remarkably reduced DENV-2 titers, as much as 2 orders of magnitude, compared to infected wild-type cells (Table 1 and Fig. 6). The fact that the No-hRz control did have reduced yields of virus can be attributed to the hygromycin selection protocol, which did impact the virus infectivity in transformed cells to some extent.

**Table 1:** Tabulation of data for TCID_50 _and qRT-PCR analyses of hRz effectiveness

	**TCID50**		**qRT-PCR Cells**		**qRT-PCR Supernatant**		
	
	Avg	SE	Avg	**SE**	Avg	SE	Avg % Red
Infected	4.39 × 10^6^	7.12 × 10^5^	1.77 × 10^6^	4.17 × 10^5^	2.92 × 10^6^	1.38 × 10^6^	0

Uninfected	2	1	3	2	3	1	N/A

No-hRz	1.88 × 10^6^	5.94 × 10^5^	1.37 × 10^6^	2.53 × 10^5^	1.60 × 10^6^	6.46 × 10^5^	41.71

Rz # 1	8.77 × 10^5^	3.4210^6^	1.62 × 10^6^	3.13 × 10^5^	9.46 × 10^5^	2.70 × 10^5^	52.03

Rz # 2	2.78 × 10^4^	1.09 × 10^4^	5.88 × 10^4^	3.95 × 10^4^	5.37 × 10^4^	1.65 × 10^4^	98.07

Rz # 3	1.16 × 10^6^	6.8910^5^	1.68 × 10^6^	3.38, × 10^5^	1.39 × 10^6^	7.62 × 10^5^	43.62

Rz # 4	2.37 × 10^6^	1.14 × 10^6^	1.99 × 10^6^	2.61 × 10^5^	1.51 × 10^6^	7.58 × 10^5^	27.39

Rz # 5	3.08 × 10^4^	9.1 × 10^3^	9.65 × 10^4^	4.90 × 10^4^	9.44 × 10^4^	2.18 × 10^4^	96.87

Rz # 6	2.33 × 10^5^	1.16 × 10^5^	1.65 × 10^6^	1.97 × 10^5^	6.45 × 10^5^	1.76 × 10^5^	59.74

Rz # 7	2.72 × 10^4^	1.13 × 10^4^	9.56 × 10^4^	2.04 × 10^4^	4.98 × 10^4^	2.49 × 10^4^	97.42

Rz # 8	1.87 × 10^5^	1.26 × 10^5^	6.46 × 10^5^	2.51 × 10^5^	4.95 × 10^5^	2.43 × 10^5^	80.76

Rz # 9	8.50 × 10^4^	3.46 × 10^4^	2.51 × 10^5^	1.24 × 10^5^	3.24 × 10^5^	1.45 × 10^5^	90.92

Rz # 10	4.07 × 10^5^	2.34 × 10^5^	5.11 × 10^5^	1.39 × 10^5^	1.36 × 10^5^	4.71 × 10^4^	85.74

Rz # 11	2.47 × 10^4^	4.01 × 10^3^	3.55 × 10^4^	1.59 × 10^4^	2.11 × 10^4^	7.41 × 10^3^	98.90

Rz # 12	5.49 × 10^5^	1.61 × 10^5^	5.51 × 10^5^	3.17 × 10^5^	3.43 × 10^5^	2.06 × 10^5^	81.54

Rz # 13	3.06 × 10^5^	1.08 × 10^5^	3.60 × 10^5^	2.11 × 10^5^	1.90 × 10^5^	1.65 × 10^5^	88.73

Rz # 14	1.98 × 10^5^	1.22 × 10^5^	2.63 × 10^5^	1.07 × 10^5^	2.63 × 10^5^	2.20 × 10^5^	90.53

Averages of results from four separate infections (Avg) are presented for each type of analysis along with Standard Error of the Mean (SE). The average percent reduction for all tests (Avg % Red) relative to the Infected cell control is calculated in the final column.

### Real-time PCR quantitation of DENV titer in whole cell RNA

The ability of each hRz to suppress DENV genome replication was quantitatively evaluated for the hRz expressing C6/36 cells using qRT-PCR to detect virus genomes in cell lysates. Total cellular RNA was extracted 7 dpi from infected hRz-transduced cells following the protocol described in Materials and Methods. First-strand cDNA was prepared using the Capsid2 primer (Table 2), followed by 40 rounds of PCR amplification using primers Capsid2 F and R. The absolute quantity of viral RNA was determined based upon comparison of infected cell Ct values against those of viral RNA standards.

**Table 2:** Designations and base sequences of twelve primer sets evaluated to select optimal primer pairs for detection of the DENV-2 NGC RNA genome

**Designation**	**Primer sequences (5'→3')**	
Capsid1	2F: caatatgctgaaacgcgaga	2R: ccatcactgttggaatcagc
Capsid2	3F: caatatgctgaaacgcgaga	3R: cgccatcactgttggaatc
Capsid3	4F: gcgagaaatacgcctttcaa	4R: ccatcactgttggaatcagc
Capsid4	5F: tatgctgaaacgcgagagaa	5R: cgccatcactgttggaatc
Capsid5	6F: gcgagaaatacgcctttcaa	6R: cgccatcactgttggaatc
Capsid6	7F: atgctgaaacgcgagagaaac	7R: ccctgctgttggtgggatt
NS51	2F: tcaaaagcattcagcacctg	2R: cacatttgggcgtaggactt
NS52	3F: gcaatgtatgccgatgacac	3R: caggtgctgaatgcttttga
NS53	4F: gcaatgtatgccgatgacac	4R: tcaggtgctgaatgcttttg
NS54	5F: tggaggagccttagtgagga	5R: acgtcccaaggttttgtcag
NS55	6F: tgagcaagaaagagggagga	6R: caggtgctgaatgcttttga
NS56	7F: caaaagcattcagcacctgaca	7R: gttaaagcgcttgcgaacct

F and R: forward and reverse primer, respectively.

**Figure 7:**
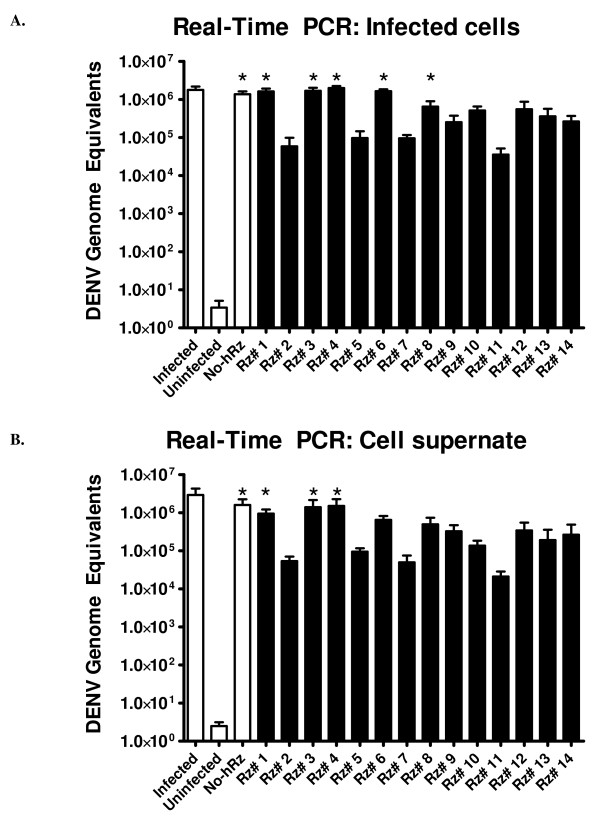
**A: Absolute quantitation of genome equivalents in cells infected with DENV and expressing hRzs**. Viral RNA samples were obtained from total cell RNA extraction at 7 dpi. ANOVA test was performed using GraphPad Prism 3.0 software. Asterisk indicate no significant differences relative to the Infected control (Dunnett's, p < 0.01). Plot is based on the average titer from 4 independent experiments. *Rz # 1–14*: 14 different infected hRz cells; *No Rz*: infected C6/36 cells transduced with the lentivirus vector lacking a hRz insert. B: Absolute quantitation of viral titers from different hRz cells. Viral RNA samples were obtained from collected cell supernatant at 7 dpi. ANOVA test was performed using GraphPad Prism 3.0 software. Asterisk indicate no significant differences relative to the Infected control (Dunnett's, p < 0.01). *Rz # 1–14*: 14 different infected hRz cells; *No Rz*: infected C6/36 cells trasnduced with the lentivurs vector lacking a hRz insert. Plots are based on the average titer from 4 independent experiments.

Four independent experiments were compared for each hRz-C6/36 transduced cell line to insure consistency and reproducibility. The results (Table 1 and Fig. 7A) for most of the hRz were consistent with the IFA determination of virus titer, with hRz-C6/36 cell lines # 2, 5, 7 and 11 exhibiting suppression of DENV replication by up to nearly 100 fold compared to the infected wild-type samples. However, the results for hRz # 6 were remarkably different from those obtained with the IFA analysis, suggesting this ribozyme may be interfering with viral genome packaging or assembly rather than replication.

### Real-time PCR quantitation of DENV titer in cellular medium

To evaluate the impact of hRzs on assembly and release of DENV, we performed qRT-PCR on the viral RNA extracted from cell supernatant of the infected hRz-C6/36 cells. Cell supernatants collected at one hour prior to whole cell RNA extraction were immediately processed for RNA extraction, cDNA synthesis and RT-PCR procedures (see Materials and Methods). The results (Table 1 and Fig. 7B) demonstrated that the titers of extracellular virus genomic RNA obtained for hRz-C6/36 cell lines # 2, 5, 7 and 11 were consistent with genome copies detected in whole cell extracts. Similar levels of reduction were seen for most of the other hRz as well. Together, these qRT-PCR findings confirmed that most of the highly effective hRzs affected viral RNA replication and not DENV assembly and release from the cells.

The levels of extracellular virion RNA for hRz # 6 were more closely related to the TCID_50 _results than to the qRT-PCR results of total cellular RNA, reinforcing the possibility that this ribozyme's effect was related to interference with virion production rather than direct suppression of viral genomes.

## Discussion

We have confirmed the effectiveness of expressed hRzs as suppressive agents of DENV in transduced mosquito cells. These ribozymes have the ability to cleave their target RNA at an NUH triplet site, and may recycle themselves provided there are short sequence homologies between the ribozyme arms and the corresponding target sequence. This could provide an advantage over standard antisense RNAs that act stoichiometrically and do not destroy the function of the targeted RNAs [28].

In this study, the ribozymes were expressed under the control of the *Ae. aegypti *tRNA^val ^promoter. Human tRNA^val ^has been used successfully to enable suppression of target genes by driving the expression of hRzs [29,30]. The tRNA^val ^utilizes RNA pol III which is involved in the transcription of short RNAs [31] and yields transcription levels 2–3 orders of magnitude greater than that of pol II systems [32]. Linking hRzs to a tRNA^val ^also enhances their activity by increasing resistance to RNases, since intracellular stability is one of the most important determinants of ribozyme efficacy [33], and facilitates the cytoplasmic transport of the attached ribozyme [25] where it can be co-localized with replicating DENV RNA.

Since intramolecular base pairing within the complex structure of the long DENV genomic RNA could preclude the association of ribozymes with their target sequences, we designed our hRz to include a 3' terminal 60 adenylic acids. This poly(A) sequence acts to recruit the unwinding activity of endogenous RNA helicase [34]. Hybrid ribozymes with this poly(A) tail are able to cleave target RNAs possessing complex secondary structure that hRz without accessory helicases cannot act upon [26,35]. In addition, the effect of helicase-attached hRzs is significantly greater than those of the conventional parental ribozymes [27].

Retroviral vectors offer a number of advantages over other gene transfer methods for the transduction of somatic cells, and have been widely used as gene delivery systems. They have the ability to stably integrate the genes they vector into host cell chromosomes with high transduction efficiencies [36-38]. Pseudotyped retroviruses displaying the vesicular stomatitis virus G glycoprotein (VSV-G) are able to transduce any dividing cells without the requirement for cell receptors [39]. This pantropic retroviral system has been successfully employed for transduction of mosquito cells [40]. In our experiments, we used self-inactivating retrovirus vectors having a defective 3' U3 that is duplicated as part of the 5' LTR during reverse transcription, allowing the hRz transgene to be expressed by the internal tRNA^val ^promoter and decreasing the possibility of promoter interference [36,41]. Transduced hRz cells were selected for hygromycin resistance for longer than 2 months. Though it is likely that less than 100% of these resistant cells were transformed, the majority of them appear to bear functional hRz vectors within the genome as indicated by the RT-PCR results of expressed hRz RNA. In addition, because these cells were transduced using lentivitus vectors, there is no real possibility that expression of the hRz came from the non-integrated vector.

Transduction of C6/36 cells by means of pantropic retrovirus vectors resulted in stable genomic integration and expression of the hRzs, rendering them persistently resistant to DENV. Since pools of transduced cells were used for the DENV challenge assays, the observed inhibition effects are not likely due to clonal variation or differing copy number of the constructs within the transduced cell population. However, this approach does allow the possibility that individual cells in the pools may be more susceptible to DENV and thus contribute to the majority of virus detected. In addition, the lower antiviral activity seen for some hRzs might also reflect a distribution effect leading to a wide range of hRz expression levels in the transduced cell cultures [17,42].

We designed 14 hRzs that target different regions along the DENV-2 genome. Quantitative real-time PCR and immunofluorescence assays demonstrate that C6/36 cells expressing certain hRzs were able to suppress DENV-2 NGC viral replication by at least 25%, with hRz-C6/36 cell lines # 2, 5, 7 and 11 having a more pronounced effect, of nearly 2 logs (100 fold) reduction in viral titer compared to the untransduced C6/36 cells. These IFA results correspond well with data obtained from the Northern hybridization analyses for those hRzs examined.

We were unable to detect the cleaved RNA products by Northern analyses due to two factors. First, there is likely rapid degradation of the DENV RNA within the cells after they are cleaved. Second, the hRz expressing cells are "armed" with many hRz molecules, and upon infection by the virus there is little chance for initiation of a productive infection during which DENV target RNA might build up to levels that cleavage products might become apparent. We interpret these results to demonstrate that suppression of virus, when it occurs, is so effective that little new viral RNA target is actually produced, and few cleavage products would be expected to be evident.

With the exception of hRz # 11, the three most active hRzs target the GUC triplet site which is cleaved most efficiently compared to other triplets [43]. GUC is frequently chosen as the target in many hRz studies because of its wide occurrence in natural hRz motifs [28]. The No-hRz transduced cells, which lack the hRz insert, exhibited no significant suppression of DENV replication, verifying that decreased levels of viral titers requires the presence of the hRz [44] and is not simply due to the presence of the transducing retrovirus or hygromycin selection.

Ng *et al*. reported 12% reduction of the *krr1 *gene expression in *Giardia canis *in cells without a hRz motif and attribute the observation to an antisense effect of the hRz forming a complex with the mRNA, inhibiting its translation and promoting its degradation [45]. While we cannot rule out an antisense effect for our hRzs, the *in vitro *analyses of ribozyme cleavage activity demonstrated that they were catalytically active. Hence, the cleavage activity of ribozymes within cellular environments would be expected to occur. The reduction in functional full-length DENV RNA was consistent with the cleavage effects of the ribozymes since the effectiveness of the hRzs we examined correlates very well with the realative effectiveness of the core cleavage site in that three of the four most effective hRzs target a GUC triplet, supporting the catalytic nature of the hRzs effectiveness and arguing against a simple antisense effect.

For the most part, the variations in relative effectiveness that we observed among the 14 hRz we analyzed can be explained by less efficient cleavage activity resulting in incomplete inhibition of DENV replication. However, there are several alternative possibilities unrelated to hRz catalysis that could influence their effectiveness. These include (i) the presence of mismatched bases in the hRz arm, especially with shorter effectors [46], due to viral escape mutants, (ii) integration of the hRz construct into an inactive gene expression region of the host cell genome, and (iii) obscuring of the target triplet site by RNA-binding proteins [28,47]. Since our experiments were done over a short time frame and with multiple replicates we can rule out the selection of escape mutations as the cause of weak suppression. Our RT-PCR analyses of the expressed hRz RNA in transformed cells did not show an appreciable difference in levels of expression, allowing us to rule out position effects on expression levels as a factor. The only possibility we cannot rule out in our experiments is the interference by RNA binding proteins at the target site.

hRz # 6 exhibited a somewhat different result from all other hRzs when comparing the relative levels of DENV-specific RNA between cellular and extracellular RNAs by qRT-PCR. The results for hRz # 6 suggest cellular DENV-specific RNA levels were not reduced to comparable levels as extracellular DENV-specific RNA levels. This implies that the reductions in virus titer observed with hRz # 6 are not related to reductions in viral RNA synthesis in infected cells, but may result from affects on packaging of the viral genomes and/or assembly and release of virions. Because hRz # 6 was not among the most effective hRzs tested, we did not pursue examination of this phenomenon.

While we were able to target several sequences that are common to all DENV serotype 2 strains, we were unable to identify suitable cleavage sites that were conserved among all DENV serotypes. Several highly conserved sequences are present among all DENV serotypes, but the requirement for an NUH triplet cleavage site makes these conserved sequences useless as targets for the hRz strategy. Other ribozymes having less stringent cleavage site requirements may be more appropriate for these conserved sequences.

Our results are comparable with results obtained from sense/antisense and siRNA strategies for suppression of DENV. dsSIN may be used in transient expression systems to validate these strategies in either cell cultures or mosquitoes [6,7] but retroviral vectors and transduced mosquito cells are more appropriate for examining the effectiveness of transgene suppression strategies in somatic cells because they allow a closer approximation of the actual endpoint of these strategies, transgenic mosquito tissues. Our results suggest the best application of this effector molecule is to constitutively express the hRz in target cells since DENV undergoes rapid RNA synthesis at 3–6 hours post infection and release of infectious virions after 12 hours. Transgenic cells pre-primed with expressed hRzs are able to act instantly in inhibiting viral replication upon entry.

Neither our hRz approach nor the siRNA strategy is wholey effective against viral escape mutants. However, one potential obstacle in using RNAi is the evidence for viral RNAi suppressors which have been reported in plants, poliovirus, and HIV-1 variants [48-50]. In the case of DENV, the presence of viral RNAi suppressors may contribute to persistent infections in which DENV is able to replicate in cells without causing cytopathic effect. hRzs are not subject to suppression by viral gene products, which could be an advantage when applied as an anti-DENV effector gene.

A number of RNA viruses exhibit significant genetic variation due to error-prone replication machinery. The simultaneous use of multiple ribozymes attacking two or more sites on the same target RNAs may be advantageous since it helps minimize loss of ribozyme activity due to base-pairing mismatches from mutation of one or the other targeted sites. However, simultaneous expression of two different ribozymes within the same transduced cell pool did not enhance the reduction of DENV titers (data not shown). Our results indicate the same levels of inhibition as obtained with the single hRz approach. This lack of synergy when two independent hRzs area employed might be caused by structural changes within the target RNA following the cleavage by one hRz that alter the cleavage domain for the second hRz, interfering with its ability to anneal with the substrate. Similar results have been reported by Xu *et al*., 1999, in which the expression of human insulin-like growth factor II is not completely inhibited by double hRzs but yields the same efficacy as single hRzs. As an alternative approach, we are currently exploring combining two hRzs as a so-called Maxizyme [25,29,30,51,52] that targets two regions with low tolerance for sequence variation, and may ultimately provide even better suppression of DENV replication in transduced mosquito cells and tissues.

Our results demonstrate that the hRz approach has significant potential as a strategy for suppressing DENV in transgenic mosquitoes, either alone or in combination with alternative genetic suppression strategies. While complete suppression of virus infectivity in infected mosquito tissues may not be possible, significant reduction of virus titers within mosquito tissues could result in a decreased efficiency of transmission for the virus, thereby reducing the prevalence of the disease among associated human populations.

## Materials and methods

### *Ae. aegypti *tRNA^val ^identification

We used the tRNA^val ^sequence identified by the fruit fly genome survey (NT_037436 REGION: complement (1342674613426818)) as a query for a BLASTN search. We limited our search to *Ae. aegypti *instances in the Genome Survey Sequence database. The search returned one hit bearing a 95% similarity (e = 5 × 10^-27^) to the *D. melanogaster *tRNA^val^, including both internal promoter sites. We then used the mFold [53] software and found the typical cloverleaf structure indicative of tRNA folding. We then added the extended stem region used by Kuwabara *et al*. [30] and analyzed the predicted folding pattern with mFold again.

### Selection of hRz target sites on DENV-2 genome

Genomic sequences of 29 strains of DENV-2 were retrieved from GenBank, aligned in ClustalX [54,55], and hRz target sites were selected by scanning for conserved sequences that contain one of the following triplets; GTC, GTA, ATC or CTC. These triplets are believed to be the most effective target sites for hRz cleavage activity [28]. The primary criterion for selection was that the target site must be present in all 29 strains of DENV-2 (DENV-2-hRz), or present only in DENV-2 NGC (DENV-2 NGC-hRz). Another important criterion for selecting suitable sites for hRz cleavage was the length of conserved flanking arms which determine the level of specificity of the ribozyme. In this study, the length of each arm ranges from 5–10 bp. The sequences of hRz templates, positions of the triplets on DENV genome, and the specific strains of DENV-2 NGC they match are listed in Table 3.

**Table 3:** Sequence, positions, and tropism of each of the 14 ribozymes used in this study

**hRz #**	**Sequence of target RNA (5'→3')**	**Target triplet and position in genome**	**DENV-2 strains targeted**
1	AGGAAAA**GUC**GUGCAAC	GUC 1325 (ENV)	NGC

2	GUUUUAG**GUC**GCCUGAU	GUC 1988 (ENV)	NGC

3	UAGCCCA**GUC**AACAUAG	GUC 2035 (ENV)	NGC

4	CAUAGGA**GUC**AUUAUCA	GUC 2323 (ENV)	NGC

5	UCACU**GUC**UGUGUC	GUC 2367 (ENV)	All strains

6	AUAGU**GUC**AUCAGUGAA	GUC 8272 (NS5)	All strains

7	AAAGAA**GUC**AGGCCA	GUC 10420 (3'NCR)	All strains

8	GCAUG**GUA**CCUGUGG	GUA 4503 (NS2B)	All strains

9	GACUCAAAA**CUC**AUGUCAGC	CUC 2974 (NS1)	All strains

10	AUGGAA**AUC**AGACCATT	AUC 3430 (NS1)	All strains

11	GGAAGCU**GUA**CGCAUGG	GUA 10528 (3'NCR)	All strains

12	UGAAGCU**GUA**GUCUC	GUA 10624 (3'NCR)	All strains

13	AUGCC**AUC**CAUGAA	AUC 10244 (NS5)	All strains

14	CUGUUGA**AUC**AACAGGU	AUC 10768 (3'NCR)	All strains

Underlined: core NUH triplet

### Plasmid construction

The hRz expression plasmid, pLAeRzARH (Figure 1), contains a given hRz-encoding gene under the control of the *Ae. aegypti *tRNA^val ^promoter. This is followed by a 60As tail that promotes hRz accessibility. A hygromycin resistance gene under control of a Rous Sarcoma Virus (RSV) promoter permits selection of transfected cells. The backbone was derived from pQCXIH retroviral vector (Clontech, Mountain View, CA). To assemble this construct, pQCXIH was digested with *Xba *I and *Eco*R V to eliminate the original IRES-hygromycin fragment from the plasmid. The RSV promoter was amplified from pLXRN (Clontech) using the following primer set; forward 5' TAATTCTAGACCGTGCGGCCGCAGATCCCCTCAGGATATAGTAGTTTC 3' and reverse 5' ACCAGATATCACGCGTGCGAAACGATCCTCATCCTGTCTCTT 3'. The PCR product was isolated from a 0.9% agarose gel using the QIAquick Gel Extraction Kit (Qiagen, Valencia, CA). The amplified RSV promoter was inserted into the digested vector at the *Xba *I and *Eco*R V sites to create pQCXR. The hygromycin resistance gene was amplified by PCR from pQCXIH using the following primers; forward 5' ATCGACGCGTATGGATAGATCCGGAAAGCCTGAACTCACCGCGACG 3' and reverse 5' ATCGACGCGTCCCCCCTTTTTCTGGAGACTAAATAAAATCTT 3'. The amplified product was purified as above and inserted into pQCXR between the *Mlu *I and *Eco*R V sites to obtain pQCXRH. pQCXRH was then cut with *Xba *I and treated with antarctic phosphatase (AP) (New England Biolabs (NEB), Ipswitch, MA) to reduce self-ligation. The 60As tail was constructed by performing a one-cycle PCR amplification using a forward primer: 5' ATCGTCTAGAAAAAAAAAAAAAAAAAAAAAAAAAAAAAAAAAAAAAAAAAAAAAAAAAAAAAAAAAAAATTTTTTACTAGTCGTA 3', which also acted as a PCR template, and a reverse primer: 5' TACGACTAGTAAAAAATTTTTTTTTTTTT 3'. The PCR product was directly purified by phenol-chloroform extraction and ethanol precipitation and double digested with *Xba *I and *Spe *I before ligating with pQCXRH linearized with *Xba *I to create pQCARH. Our novel *Ae. aegypti *tRNA^val ^promoter gene was synthesized from two oligonucleotides using Klenow large fragment DNA polymerase (NEB) and a pair of primers; forward – 5' ATCGTCTAGACCGTTGGTTTCCGTAGTGTAGTGGTTATCACGTCTGCTTCACACGCAGAAGGTCCCCGGTTCGAACCC 3' and reverse – 5' ATCGTCTAGACGATCAATTGCAGTCTCGAGAAAGTTTGGTTTTTGTAGTGCCCGGGTTCGAACCGGGGACCTTCTGCG 3'. The amplified product was purified as above, digested and ligated into pQCARH at the unique *Xba *I site. To achieve higher packaging efficiency, the entire insert spanning the *Ae. aegypti *tRNA^val ^promoter to the hygromycin resistance gene was inserted into pLXRN (Clontech). The RSV-neomycin resistance gene originally present in pLXRN was eliminated by digestion with *Sph *I and *Csp*45 I, followed by end-filling with Klenow fragment and ligation. To obtain pLAeARH, the region from *Ae. aegypti *tRNA^val ^promoter to the hygromycin resistance gene from pQAeARH was amplified by PCR using the following primer set; forward 5' ATTAGGATCCTCTAGACCGTTGGTTTCCGTAGTG 3' and reverse 5' ATTACCTAGGGATATCCTGTCTTTAACAAATTGGACTAATC 3' and inserted into the modified pLXRN plasmid using *Avr *II and *Bam*H I sites. hRz genes were then inserted between *Csp*45 I and *Mfe *I sites. hRz templates (Table 4) were amplified using the following set of primers; forward 5' ATCGACGCGTATGGATAGATCCGGAAAGCCTGAACTCACCGCGACG 3' and reverse 5' GATGAATTCAAAAAAACAATTGT 3'. The PCR product was purified as above and cut with *Csp*45 I and *Mfe *I before insertion into pLAeARH, generating pLAeRzARH. Restriction analysis and DNA sequencing were performed after each cloning step to confirm correct base sequences. All plasmids were purified by CsCl_2 _gradient centrifugation.

**Table 4:** Sequences of the fourteen ribozyme templates used for PCR amplification

**hRz #**	**Template sequence for amplification (5' to 3')**
1	CCGGTTCGAACCCGGGCACTACAAAAACCAACAAgttgcac**CTGATGAGGCCGAAAGGCCGAAA**cttttcctACAATTGTTTTTTTGAATTCATC

2	CCGGTTCGAACCCGGGCACTACAAAAACCAACAAatcaggc**CTGATGAGGCCGAAAGGCCGAAA**cctaaaacACAATTGTTTTTTTGAATTCATC

3	CCGGTTCGAACCCGGGCACTACAAAAACCAACAActatgtt**CTGATGAGGCCGAAAGGCCGAAA**ctgggctaACAATTGTTTTTTTGAATTCATC

4	CCGGTTCGAACCCGGGCACTACAAAAACCAACAAtgataat**CTGATGAGGCCGAAAGGCCGAAA**ctcctatgACAATTGTTTTTTTGAATTCATC

5	CCGGTTCGAACCCGGGCACTACAAAAACCAACAAgacaca**CTGATGAGGCCGAAAGGCCGAAA**cagtgaACAATTGTTTTTTTGAATTCATC

6	CCGGTTCGAACCCGGGCACTACAAAAACCAACAAttcactgat**CTGATGAGGCCGAAAGGCCGAAA**cactatACAATTGTTTTTTTGAATTCATC

7	CCGGTTCGAACCCGGGCACTACAAAAACCAACAAtggcct**CTGATGAGGCCGAAAGGCCGAAA**cttctttACAATTGTTTTTTTGAATTCATC

8	CCGGTTCGAACCCGGGCACTACAAAAACCAACAAccacagg**CTGATGAGGCCGAAAGGCCGAAA**ccatgcACAATTGTTTTTTTGAATTCATC

9	CCGGTTCGAACCCGGGCACTACAAAAACCAACAAgctgacat**CTGATGAGGCCGAAAGGCCGAAA**gttttgagtcACAATTGTTTTTTTGAATTCATC

10	CCGGTTCGAACCCGGGCACTACAAAAACCAACAAaatggtct**CTGATGAGGCCGAAAGGCCGAAA**tttccatACAATTGTTTTTTTGAATTCATC

11	CCGGTTCGAACCCGGGCACTACAAAAACCAACAAccatgcg**CTGATGAGGCCGAAAGGCCGAAA**cagcttccACAATTGTTTTTTTGAATTCATC

12	CCGGTTCGAACCCGGGCACTACAAAAACCAACAAgagac**CTGATGAGGCCGAAAGGCCGAAA**cagcttcaACAATTGTTTTTTTGAATTCATC

13	CCGGTTCGAACCCGGGCACTACAAAAACCAACAAttcatg**CTGATGAGGCCGAAAGGCCGAAA**tggcatACAATTGTTTTTTTGAATTCATC

14	CCGGTTCGAACCCGGGCACTACAAAAACCAACAAacctgtt**CTGATGAGGCCGAAAGGCCGAAA**ttcaacagACAATTGTTTTTTTGAATTCATC

Bold uppercase: conserved catalytic core. Lowercase: 5' and 3' arms of the hRz, respectively.

### Retroviruses

Retroviruses encoding the 14 hRzs and No-hRz control were produced following established methods [56]. Briefly, the retroviruses were assembled by simultaneous transfection of 293FT cells (Invitrogen, Carlsbad, CA) with the pLAeRzARH (5 μg) and plasmids encoding murine leukemia virus gag-pol (5 μg) and the vesicular stomatitis virus (VSV) envelope protein (2 μg). The VSV envelope protein confers broad host range and allows high efficiency transduction of the transgene into *Aedes *cells [39]. After 48 hr, culture supernatants containing the retroviruses were collected and filtered through a 0.45 um filter to remove cell debris. Virus was then applied to target cells or stored frozen at -80°C until required.

### Cell Culture

#### Maintenance

*Ae. albopictus *(C6/36) mosquito cells used in this study were maintained in complete L-15 (Atlanta Biologicals) with 10% FBS (Atlanta Biologicals), 10% tryptose phosphate broth (Sigma), and 1% antibiotic/antimycotic solution (Sigma). hRz transduced cells were maintained in complete L-15 with 35 μg/mL hygromycin (Invitrogen). For infection of cells, complete L-15 without any antibiotics was used. Cells were incubated at 28°C without CO_2_.

#### Infection with retroviruses and selection of transduced cells

Wild-type C6/36 cells in 6-well plates were infected with retroviruses at an MOI of 30. After 24 hr, the medium was replaced and the cells were allowed to grow for one week before being transferred to T25 flasks. Complete L-15 medium with hygromycin B was added to the T25 flasks after 72 hr to select stably transduced cells.

#### Infection with DENV-2 NGC

Wild-type C6/36 cells were seeded at a density of 0.5 × 10^6 ^cells/mL in 6-well plates and incubated for 2 hr at 28°C to allow attachment. Once attached, cells were washed twice with medium and challenged with DENV-2 NGC stock at an MOI of 0.01. Infection was allowed to continue for 7 days at 28°C before viral RNA was extracted both from cells and cultured supernatants using TRI reagent (Invitrogen), and purified QIAamp Viral RNA Mini Kit (Qiagen), respectively.

### Northern analysis

Total RNA was extracted using TRI reagent (Invitrogen), and 10 μg was loaded on 1% denaturing agarose gels. RNA was transferred overnight onto nylon membranes, and the membranes were fixed by UV cross linking, prehybridized in Church Buffer (67 g Na_2_HPO_4_, 2 ml H_3_PO_4_, 70 g SDS in 1 L distilled water; filter sterilized before use) for an hour, and then hybridized with specific ^32^P-labeled probe overnight in a 65°C oven. The membranes were washed 2× with washing solution 1 (2× SSC, 0.1% SDS), and another 2× with washing solution 2 (0.2× SSC, 0.1% SDS), each wash at 65°C for 25–30 minutes. The DNA probes were designed to detect different regions along the virus genome and were synthesized using Prime-A-Gene Kit (Promega, Madison, WI). Unincorporated unlabelled nucleotides were removed by QIAquick Gel Extraction Kit (Qiagen) before use. The β-actin gene was used as an internal control for RNA extraction and hybridization. After washing, the autoradiograph was exposed for 6–16 hours before developing. The autoradiograph was scanned and the relative amount of viral RNA in each sample was determined by densitometry using ImageJ Java image processing software . Measurements for each sample were normalized to that of infected wild-type cells to determine the relative virus replication efficiency.

### *In vitro *analysis of hRz cleavage activity

The procedure for *in vitro *hammerhed ribozyme cleavage reactions was adapted from Shao Y. *et al *[57]. Two complementary oligonucleotides containing the hRz catalytic core sequence flanked by the sequences of the two binding arms and a T7 promoter sequence were synthesized (Invitrogen) and annealed together in equimolar concentrations using the following conditions: 10 mM Tris-HCl pH 8.0, 50 mM NaCl. The reaction was heat denatured at 95°C for 5 min followed by slow cooling to room temperature. The annealed templates were cloned into the pET11a plasmid (Novagen) and linearized with *Cla *I for *in vitro *run-off transcription.

Primer sets were used to PCR amplify a 1370 bp target spanning the 1497–2866 bp envelope region (ENV forward – 5' TTTTAAAATTCTAGAGAGAACGGGCCTCGACTT 3', and reverse – 5' TTTAAATTTAGATCTCTCTGTTTGTGTTGGGGCATT 3') and a 940 bp target spanning the 9774–10713 bp 3'NCR (NCR forward – 5' AAATTAAAATCTAGAAGTTCCATGCAGAAACCAAG 3', and reverse – 5' ATTTTATTTAGATCTGATTCAACAGCACCATTCCA 3') from the DENV-2 NGC Infectious Clone vector. The envelope region contains hRzs # 2 and 5 sites while the 3'NCR region contains hRzs # 7 and 11 sites. The amplified fragments were digested with *Xba *I and *Bgl *II and cloned into the *Xba *I and *BamH *I-digested pET11a vector.

2.5 ug of linearized ribozyme plasmid and 1.5 ug of ribozyme template were *in vitro *transcribed using the Megashortscript and Megascript (Ambion, USA), respectively. *In vitro *transcription reactions were performed following the manufacturer's protocol with incubation for 4 hr at 37°C. The transcripts were phenol: choloroform: isoamylalcohol (24:25:1) extracted, choloroform: isoamyalcohol (24:1) extracted and ethanol precipitated with 0.2 ug of glycogen. The RNA concentration was measured spectrophotometrically.

*In vitro *transcribed targets (10 pM) were mixed with 50 pM of ribozymes in 50 mM Tris-HCl, pH-8.0, heat denatured at 85°C for 3 min, and snap cooled in ice. The cleavage reaction was initiated by addition of 20 mM MgCl_2_, and 40 U of RNase inhibitor and incubated at 37°C for 30 min. Loading buffer II (Ambion) was added and the reaction mixture was incubated at 65°C for an additional 5 min followed by electrophoresis in a 2% agarose/formaldehyde/MOPS gel [57] for 1 hr at 105 V. After electrophoresis the gel was stained with ethidium bromide and photographed with UV illumination.

### TCID_50 _immunofluorescence assay

Supernatants containing virus from infected cells were collected 4 dpi and were 10-fold serially diluted in a 96-well plate. C6/36 cells from a confluent flask were then added to the mix and the cultures were incubated for 4 days at 28°C without CO_2_. After incubation, cells were fixed with acetone:DPBS (3:1) and stained with primary antibody (1:200) specific to the envelope (ENV) protein of DENV [58]. Detection of positive DENV-infected cells was accomplished by utilizing a biotinylated-secondary antibody and streptavidin detection system (Amersham Biosciences, Piscataway, NJ). Cells that showed fluorescence within the cytoplasm were scored as positive for DENV infection. The TCID_50 _was calculated based on Karber's method [59]. The titer was expressed as log_10_TCID50/0.1 ml.

### Real-Time PCR

#### Run mode and machine

All absolute quantitation experiments in this study were performed under the 9600 Emulation run mode of AB7500 Fast machine from Applied Biosystems (Foster City, CA). The PCR profile was 1 cycle of 50°C for 2 min, 1 cycle of 95°C for 10 min and 40 cycles of 95°C for 15 sec and 60°C for 1 min. Dissociation curve analysis was added to the end of each run. This was initiated by heating the PCR product to 95°C and holding it for 15 sec. Then the denatured DNA was allowed to anneal by ramping down to 60°C for 1 min. The temperature was then slowly ramped up again to 95°C for 15 sec and fluorescence signal was collected during this time. Once the temperature reached 95°C, the PCR run was terminated. Results were analyzed using the company's Sequence Detection Software Version 1.3.

#### Determination of hRz retroviral titers

Retroviral RNA was extracted using the QIAamp Viral RNA Mini Kit (Qiagen) following the manufacturer's protocol without any modification. The primers used to detect retroviral transcripts were specific to a region corresponding to the packaging signal of the retrovirus. Standard curves were generated based upon known titers of a control virus, pLeGFP, for which the titer was determined by counting GFP expressing cells in a limiting dilution assay (3 × 10^6 ^colony forming units per ml).

#### Evaluation and selection of the primer set used for detection of DENV-2 NGC RNA

Viral RNA was extracted from infected C6/36 cells 7 dpi using QIAamp Viral RNA Mini Kit (Qiagen). Twelve sets of primers were evaluated for detection of the capsid and NS5 regions of the DENV-2 NGC RNA viral genome (Table 2). All primers were designed using the Primer3 freeware website . An optimal primer pair was chosen based on a standard curve (slope value ranges within -2.8 to -3.2 and R^2 ^value ranges within 0.970 to 0.999) and a single specific peak on a dissociation curve generated after the PCR run. Slope values of -3.2 indicated 100% amplification efficiency and R^2 ^values of 1.000 suggested the perfect fit of standard curve data to a straight line. Dissociation curves were plotted after the run to assure the specificity of the PCR product. Higher peaks displayed at T_m _of 80°C indicated a specific PCR product while sub peaks at a lower T_m _represented primer-dimers and non-specific PCR products. The primer pair, forward 5' CAATATGCTGAAACGCGAGA 3' and reverse 5' CGCCATCACTGTTGGAATC 3', was chosen for further experiments as it offered the highest amplification efficiency and gave a single specific PCR product.

#### Preparation of DENV-2 standard curve

Wild-type C6/36 cells were infected with the DENV-2 NGC strain and viral supernatants were collected 7 dpi. Virus titers were determined by tissue culture infectious dose 50% (TCID_50_) immunofluorescence assays using a primary antibody against the virus envelope. Viral RNA was extracted as described above and cDNA was synthesized through reverse transcription with MuLV reverse transcriptase (Applied Biosystems) and 5-fold serially diluted for generation of the standard curve.

#### Preparation of viral RNA samples

Viral RNA from infected cells was extracted using the TRI reagent (Invitrogen). RNA concentrations were determined spectrophotometrically. Viral RNAs were extracted from collected supernatants as mentioned above. cDNAs of each sample were synthesized as described above, and the titers of DENV from each sample were determined based on a standard curve. The presence of a single specific PCR product was also confirmed via a dissociation curve.

### Statistics

ANOVA test was performed using GraphPad Prism 3.0 software. Means were considered statistically significant when p-values less than 0.01 were obtained with the Dunnett's post-test. Lack of statistical significance with regards to Infection control is indicated on all figures as asterisks above bars.

## Competing interests

The authors declare that they have no competing interests.

## Authors' contributions

PN was responsible for design and completion of the bulk of the research, as well as for data analysis and initial writing of this report. JK was responsible for identification of the *Ae. aegypti *tRNA^val^, and contributed critical thinking and planning towards the development of the ribozymes and clones used in the study. TF was responsible for facilitating all cell culture aspects of the research, including establishing, selecting, and maintaining all transduced cell lines. AK and RD were instrumental in providing support to the research in the form of both instruction in and preparation of all lentivirus vectors used in this report. SH was responsible for training and contribution of antibodies and methodologies for all DENV *in vitro *analyses, and AM contributed expertise towards the Northern blot analyses. VB was responsible for the *in vitro *ribozyme cleavage analyses. YR and NK were instrumental in securing funding from the Thailand Research Fund and Faculty of Tropical Medicine, Mahidol University, respectively in support of PN. MJF was the Principal Investigator and is primarily responsible for all aspects of the funding, research design, interpretation, and writing of this manuscript. This research was supported by NIH/NIAID RO1 AI48561 to MJF and by the Royal Golden Jubilee Ph.D. program PHD/0175/2543 from the Thailand Research Fund to PN and YR.
